# Severe and Fatal Rocky Mountain Spotted Fever After Exposure in Tecate, Mexico — California, July 2023–January 2024

**DOI:** 10.15585/mmwr.mm7347a1

**Published:** 2024-11-28

**Authors:** Anne M. Kjemtrup, Jill K. Hacker, Madeleine Monroe, Vicki Williams, Charles Lines, Karla Lopez, Christopher D. Paddock, Ann Carpenter, Johanna S. Salzer, Julian A. Villalba, Julu Bhatnagar, Seema Shah, Esmeralda Iniguez-Stevens, Theodore C. Efthemeou, Vannia Hernandez, Duc J. Vugia, Vicki L. Kramer

**Affiliations:** ^1^Infectious Diseases Branch, California Department of Public Health; ^2^Viral and Rickettsial Disease Laboratory, Center for Laboratory Sciences, California Department of Public Health; ^3^County of San Diego Health and Human Services Agency, San Diego, California; ^4^County of San Bernadino, Department of Public Health, San Bernadino, California; ^5^Imperial County Public Health Department, El Centro, California; ^6^Division of Vector-Borne Diseases, National Center for Emerging and Zoonotic Infectious Diseases, CDC; ^7^Epidemic Intelligence Service, CDC; ^8^Division of High Consequence Pathogens and Pathology, National Center for Emerging and Zoonotic Infectious Diseases, CDC; ^9^Office of Binational Border Health, California Department of Public Health, San Diego, California.

**Summary**

**What is already known about this topic?**

Rocky Mountain spotted fever (RMSF) is a tickborne disease endemic in areas of the Americas. Persistent high incidence of the disease exists in northern Mexico, perpetuated by local populations of brown dog ticks (*Rhipicephalus sanguineus* sensu lato) and free-roaming dogs.

**What is added by this report?**

During July 2023–January 2024, six cases of RMSF in persons with exposure in Tecate, Mexico were reported to the California Department of Public Health; three patients died. This outbreak highlights a newly recognized location in Baja California with high RMSF risk.

**What are the implications for public health practice?**

Increased awareness of RMSF among health care providers on both sides of the border between the United States and Mexico would facilitate prompt treatment and help prevent fatalities.

## Abstract

Rocky Mountain spotted fever (RMSF) is a tickborne disease endemic in areas of the Americas. Persistent high incidence of the disease exists in northern Mexico, perpetuated by local populations of brown dog ticks (*Rhipicephalus sanguineus* sensu lato) and free-roaming dogs. Six cases of RMSF caused by *Rickettsia rickettsii*, including three deaths, were reported to the California Department of Public Health during July 2023–January 2024. All six patients were eventually determined to have had exposure to *R. rickettsii* in Tecate, Mexico, a municipality on the U.S. border that had not been previously described as a high-risk RMSF area. Identification and reporting of the cases were complicated by challenges in diagnosis. The serious nature of the disease and delays in initiating appropriate treatment can result in life-threatening consequences. Epidemiologic collaborations among local, state, federal, and international public health agencies were essential to identifying Tecate as the location of exposure. Further collaborations will be important for directing future prevention measures. Increased health care provider awareness of RMSF is critical on both sides of the border to facilitate earlier diagnosis and initiation of appropriate treatment.

## Investigation and Results

### Identification of Cases

Cases of Rocky Mountain spotted fever (RMSF), a life-threatening tickborne disease caused by *Rickettsia rickettsii*, are reported electronically to the California Department of Public Health (CDPH) from commercial laboratories or county health departments. Local health departments investigate cases collaboratively with agencies including CDPH, local vector control, and CDC. In October 2023, CDPH was notified of *R. rickettsii* detection using a Karius Test (Karius, Inc.), a microbial cell-free DNA (mcf DNA) assay on a whole blood specimen from a patient with fatal suspected RMSF. The CDPH-Viral and Rickettsial Disease Laboratory (VRDL) confirmed the result by real-time reverse transcription–polymerase chain reaction (RT-PCR) testing (*1*). One week later, a second fatal case of RMSF was identified by RT-PCR at the CDPH-VRDL, and formalin-fixed, paraffin-embedded (FFPE) tissue tested positive by PCR at CDC (*2*). Additional cases were identified retrospectively through inquiries to local border county hospitals and prospectively through CDPH communication with California public health officials. This activity was reviewed by CDC, deemed not research, and was conducted consistent with the applicable federal law and CDC policy.*

### Patient Characteristics

The six RMSF patients ranged in age from 17 months to 65 years, and all but one were male (Table). Three of the patients were U.S. residents. Interviews with surviving patients or their family members revealed that each patient had traveled to or lived in Tecate, Mexico within 8 days of illness onset. All had exposure to dogs in Tecate; one patient reported a tick bite. No patients had known relationships with one another, except for patients B and C described below.

**TABLE:** Characteristics of six patients who received diagnoses of Rocky Mountain spotted fever with exposure in Tecate, Mexico — California, 2023–2024

Characteristic	Patient					
	A	B	C	D	E	F
**Age, sex**	17 mos, M	4.5 yrs, M	3 yrs, M	65 yrs, M	17 yrs, F	13 yrs, M
**Onset mo, yr**	Jul 2023	Aug 2023	Aug 2023	Oct 2023	Oct 2023	Jan 2024
**Symptom/Treatment, no. of days from onset/Action**						
Onset of cutaneous manifestations	2	1 (approx.)	1 (approx.)	7	12 (approx.)	3
Start of doxycycline treatment	10	NA	5	7	10	6
Sample collection*	10, a	5, a	5, a 6, b	5, a	9, a 12, b 15, c postmortem, d	6, a
Sample collection*/Laboratory results* (testing location)^†^	a Karius pos (comm.); RT-PCR pos IgG = 1:256 (VRDL)	a PCR pos Molecular evidence of *R. rickettsii* in kidney, liver, and other tissues; IHC evidence *Rickettsia* sp. spleen and other tissues. (CDC)	a IgG neg (comm.); b Karius pos (comm.); RT-PCR pos (VRDL)	a IgG = 1:64 (comm.); RT-PCR pos IgG = 1:2,048 (VRDL)	a Karius pos (comm.); RT-PCR pos (VRDL) b IgG = 1:128 (comm.); c IgG = 1:512 (comm.); RT-PCR neg (VRDL) d Molecular evidence of *R. rickettsii* in liver; IHC evidence of spotted fever group *Rickettsia* sp. (CDC)	a IgM = 1:128 IgG neg (comm.); Karius pos (comm.); RT-PCR pos (VRDL)
**Outcome**	Survived	Died	Survived	Died	Died	Survived
**Exposure in Tecate; for nonresidents, no. of days before onset**	Visited Tecate for 14 days when illness began	Tecate resident	Tecate resident	7	8 (approx.)	Tecate resident
**Classification**	Confirmed	Confirmed	Confirmed	Confirmed	Confirmed	Confirmed

**Abbreviations:** approx. = approximately; comm. = commercial laboratory; F = female; IgG = immunoglobulin G; IgM = immunoglobulin M; IHC = immunohistochemistry; M = male; NA = not available; neg = negative; pos = positive; RT-PCR = reverse transcription–polymerase chain reaction; VRDL = Viral and Rickettsial Disease Laboratory.

* Order of sample collections indicated by letters a–d: a = first, b = second, c = third, and d = fourth.

^†^ All RT-PCR tests were conducted at the California Department of Public Health, VRDL; the Karius Test is a proprietary commercial microbial cell-free DNA metagenomic test performed by Karius, Inc. laboratories.

Patient A, a child aged 17 months, was evaluated in a Tecate clinic in July 2023 for fever and presumed gastroenteritis and received amoxicillin-clavulanic acid on the second day of illness. He vomited after 2 doses of antibiotics, and 1 day later developed a rash. The following week, he was seen in an emergency department (ED) in San Diego, California, where differential diagnoses included viral exanthem and a drug reaction. On illness day 7, he was taken again to a clinic in Tecate and was prescribed a cephalosporin antibiotic, which he tolerated; however, his symptoms did not abate. On day 10, he became lethargic and difficult to arouse and was taken to a tertiary care facility in San Diego. At the San Diego facility, a petechial rash on palms and soles of feet and on oral mucosa prompted consideration of a diagnosis of bacterial meningitis or rickettsial disease, and antibiotic coverage was broadened to include doxycycline. Blood was sent to Karius laboratories where *R. rickettsii* mcf DNA was detected; the diagnosis was later confirmed at CDPH-VRDL by RT-PCR (Table). After >2 weeks of hospitalization, the patient recovered. Exposure included visiting Tecate, where his family reported large numbers of ticks, dogs, and other apparent human cases of RMSF-like illness in the community.

**FIGURE 1:**
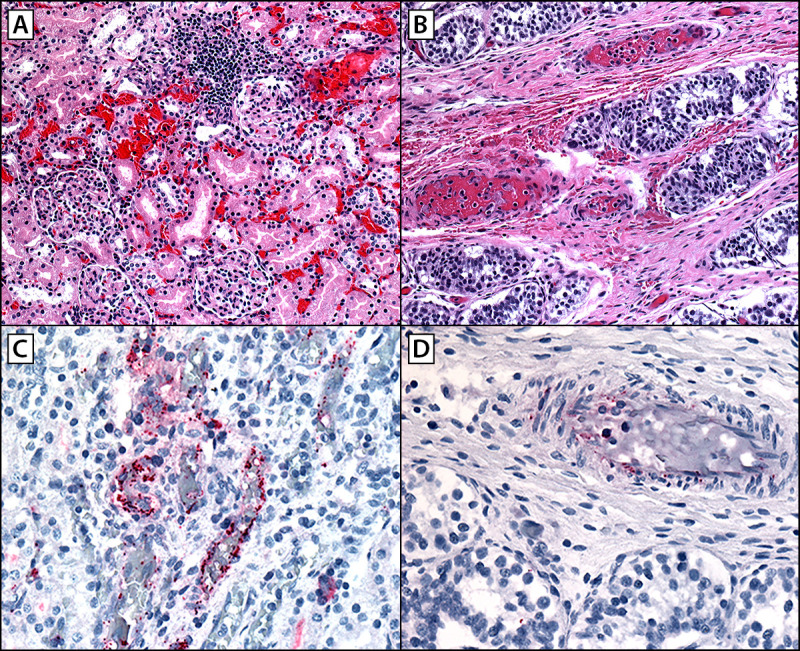
Histopathologic findings in a fatal pediatric case of Rocky Mountain spotted fever indicating extensive microhemorrhages, vascular inflammation, and endothelial injury in multiple organs including kidneys (A), testes (B), and rickettsial antigens identified by immunohistochemistry distributed predominately in endothelial cells of capillaries, arterioles, and venules in the vasa recta in the kidneys (C), and in interstitial areas in the testes (D) — California, August 2023

**FIGURE 2:**
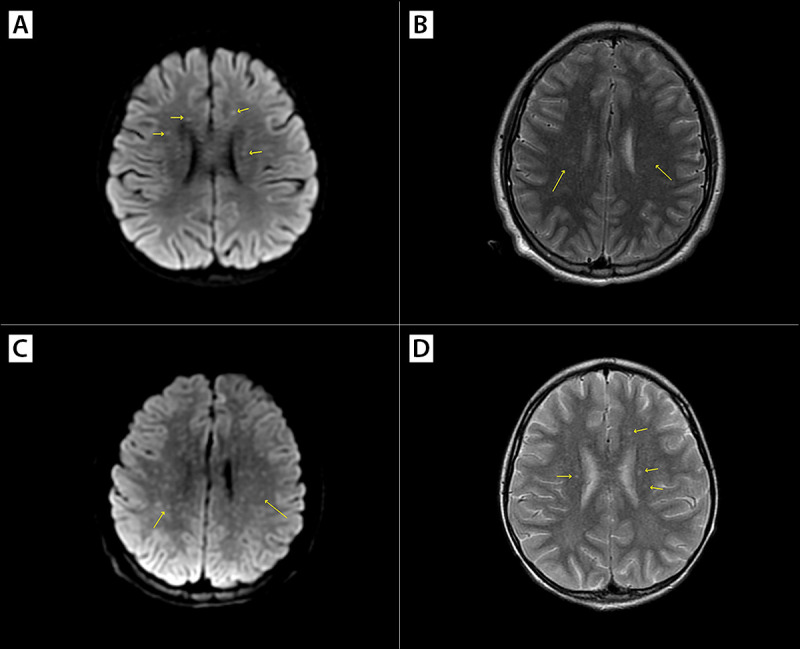
Magnetic resonance imaging findings*^,^^†^ for cases of Rocky Mountain spotted fever in two residents of Tecate, Mexico (a child aged 3 years [A and B] and an adolescent aged 13 years [C and D]) with small foci of signal abnormality in the white matter indicated (“starry sky” appearance)^§^ — California, August 2023 * Axial diffusion-weighted imaging, panels A and C. ^†^ Axial T2-weighted imaging, panels B and D. ^§^ Multiple punctate foci of reduced diffusivity and edema, suggesting white matter ischemic foci associated with vasculitis.

Patients B and C were siblings aged 4.5 and 3 years, respectively, who became ill on the same day in August 2023 in Tecate. A tick was removed from patient C at home 2 days before symptom onset; when the tick attached was not known. Both children developed a rash, initially thought to be varicella, 2 days after the tick was found and removed from patient C, and both became lethargic over the next 2 days. Patient B developed diarrhea and respiratory difficulty, and the rash on both children spread to involve the whole body. Health care was sought in California on the fifth day of illness; patient B succumbed to cardiac failure en route to the health care facility. RT-PCR testing of a postmortem blood specimen returned a positive result for *R. rickettsii*. FFPE tissue from multiple organs (skin, kidney, liver, spleen, thymus, testis, adrenal gland, and tongue) obtained at autopsy and evaluated at CDC’s Infectious Diseases Pathology Branch indicated extensive, predominantly small-vessel, vasculitis and abundant antigens of spotted fever group *Rickettsia* when stained using immunohistochemistry (IHC) (Figure 1). DNA extracts from IHC-reactive FFPE tissue blocks were positive for *R. rickettsii* by PCR. Patient C was taken to a tertiary care facility in San Diego, where treatment with doxycycline was started in the ED because of a high index of suspicion for RMSF; the diagnosis was confirmed 2 days later by a Karius Test positive for *R. rickettsii* mcf DNA. Patient C’s evaluation in San Diego included magnetic resonance imaging of the brain, which showed small foci of signal abnormality in the white matter (known as a “starry sky” appearance), a finding highly associated with RMSF (Figure 2).

Photos/Infectious Diseases Pathology Branch, CDC

Photos/County of San Diego Health and Human Services Agency, San Diego, California

Patient D, a man aged 65 years, was evaluated in a San Diego ED in October 2023 after 4 days of abdominal pain with fever, malaise, and body aches. Severe thrombocytopenia prompted concern for thrombotic thrombocytopenic purpura, and he was admitted to an intensive care unit. A diffuse petechial rash developed on day 7 of illness, prompting administration of doxycycline because of suspicion of RMSF. Respiratory failure ensued, and the patient died on the same day. A family interview revealed that he had traveled to a rural area of Tecate 1 week before illness onset and had noted the presence of free-roaming dogs.

Patient E, an adolescent girl aged 17 years, was seen at a California ED in October 2023 after 4 days of a sore throat, myalgias, and headaches. A drop in blood pressure and the onset of abdominal pain while seeking care with a primary care provider prompted admission to the hospital where her illness was determined to be consistent with sepsis. Despite broad antibiotic coverage (not including doxycycline), she developed respiratory failure and a brain injury. A Karius Test ordered on day 9 of illness was positive for *R. rickettsii* mcf DNA, and doxycycline was added to the treatment. A scattered petechial and papular rash over her extremities was identified on day 15 of illness, and she died on day 16. No ticks were found near her home in California; family interviews revealed that the patient had traveled to Tecate 8 days before illness onset.

Patient F, an adolescent boy aged 13 years, became ill with cough and fever in January 2024 while in Tecate. His family reported a small red bump on his arm 1 week earlier that developed into a dark scab. A rash appeared over a majority of his body 2 days later. Health care was sought at an ED in San Diego. He was started on doxycycline because of suspicion of RMSF. A magnetic resonance imaging of the brain demonstrated a small foci of signal abnormality in the white matter (Figure 2). After a 35-day hospitalization, he completed 2 weeks of rehabilitation before discharge.

### Public Health Response

This outbreak of severe and fatal RMSF after exposures in Tecate, Mexico prompted communications among CDPH, CDC, and Baja California public health officials, who confirmed that the number of RMSF cases was increasing in Tecate. To increase awareness among health care providers and travelers regarding RMSF and the risk posed by exposure to dogs and ticks in northern Mexico, various health alerts were issued: a Health Alert Network (HAN) health advisory in San Diego County (November 3, 2023), a National HAN Advisory (December 8, 2023), and a Travel Health Notice (March 12, 2024). In addition, a coordinated multinational health care provider education activity for approximately 200 health care workers at the two principal hospitals in Tecate was coordinated by the Instituto de Servicios de Salud Pública del Estado de Baja California (Institute of Public Health Services of the State of Baja California) and CDC in May 2024 to provide information on the emerging risk, diagnosis, and treatment of RMSF. Since January 2024, no additional cases with exposure in Tecate, Mexico have been reported in California; one case with exposure in Mexicali, Mexico has been reported.

## Discussion

Rocky Mountain spotted fever is a severe tickborne disease endemic in the Americas. Since the beginning of the 21st century, hyperendemic^†^ levels of RMSF have emerged on tribal lands in the southwestern United States and across multiple states of northern Mexico (*3*,*4*), including several border cities of Baja California (*5*,*6*), where the brown dog tick (*Rhipicephalus sanguineus* sensu lato) serves as the principal vector.

The identification of six confirmed cases of RMSF in southern California over 6 months is unusual; during 2011–2019, an average of one confirmed RMSF case per year was reported for the state of California (*6*). Because all patients included in this report had lived in or traveled to Tecate, Mexico within the 14-day RMSF incubation period, this investigation highlights the risk for RMSF along the border region. Vector-control inspection of patients’ homes in California was important to identify whether exposure was local or occurred in Mexico or elsewhere, as described for other California cases (*7*). Fatal RMSF cases acquired in Mexico have been documented in other U.S. border states, including Arizona (*8*). Cross-border collaboration and communication among health authorities to effectively monitor, report, and respond to this disease are critical (*3*).

The clinical and diagnostic challenges observed during treatment for these patients underscore the challenges associated with diagnosing RMSF, particularly in localities where RMSF is rarely encountered (*9*). Initial signs and symptoms, including fever, cough, or abdominal pain, can mimic those of other diseases. If RMSF is suspected, treatment should begin immediately because there are no rapid, point-of-care diagnostic tests to confirm acute disease. Molecular testing, either targeted RT-PCR (*1*) or metagenomic testing, have improved diagnostic sensitivity during the acute phase of disease; the Karius Test was essential for the diagnosis or confirmation of RMSF cases among four of these patients. However, the rapid clinical progression of RMSF from a moderately severe illness to a life-threatening disease necessitates early initiation of doxycycline, as soon as the disease is suspected clinically, without waiting for confirmation of the diagnosis (*10*).

### Implications for Public Health Practice

This outbreak highlighted a new area of RMSF risk in Mexico and underscored the need for health care provider awareness on both sides of the U.S.-Mexico border to treat suspected RMSF patients quickly with doxycycline to reduce risk for death. Collaborative activities among local, state, and international health agencies were necessary for determining exposure and establishing diagnosis for some of these patients. Continued binational collaborations on surveillance and communication will be important for future prevention measures.

## Footnotes

* 45 C.F.R. part 46,21 C.F.R. part 56; 42 U.S.C. Sect. 241 (d); 5 U.S.C. Sect. 552a; 44 U.S.C. Sect. 3501 et seq.

† Hyperendemic RMSF is defined as persistence of regional foci of disease in locations where the annual incidences consistently exceed the national annual incidence.
